# The Rho GTPase Cell Division Cycle 42 Regulates Stereocilia Development in Cochlear Hair Cells

**DOI:** 10.3389/fcell.2021.765559

**Published:** 2021-10-22

**Authors:** Haibo Du, Hao Zhou, Yixiao Sun, Xiaoyan Zhai, Zhengjun Chen, Yanfei Wang, Zhigang Xu

**Affiliations:** ^1^Shandong Provincial Key Laboratory of Animal Cell and Developmental Biology, School of Life Sciences, Shandong University, Qingdao, China; ^2^State Key Laboratory of Cell Biology, Center for Excellence in Molecular Cell Science, Shanghai Institute of Biochemistry and Cell Biology, Chinese Academy of Sciences, Shanghai, China; ^3^School of Life Sciences and Technology, ShanghaiTech University, Shanghai, China; ^4^Shandong Provincial Collaborative Innovation Center of Cell Biology, Shandong Normal University, Jinan, China

**Keywords:** inner ear, hair cells, stereocilia, CDC42, knockout mice

## Abstract

Stereocilia are actin-based cell protrusions on the apical surface of inner ear hair cells, playing a pivotal role in hearing and balancing sensation. The development and maintenance of stereocilia is tightly regulated and deficits in this process usually lead to hearing or balancing disorders. The Rho GTPase cell division cycle 42 (CDC42) is a key regulator of the actin cytoskeleton. It has been reported to localize in the hair cell stereocilia and play important roles in stereocilia maintenance. In the present work, we utilized hair cell-specific *Cdc42* knockout mice and CDC42 inhibitor ML141 to explore the role of CDC42 in stereocilia development. Our data show that stereocilia height and width as well as stereocilia resorption are affected in *Cdc42*-deficient cochlear hair cells when examined at postnatal day 8 (P8). Moreover, ML141 treatment leads to planar cell polarity (PCP) deficits in neonatal hair cells. We also show that overexpression of a constitutively active mutant CDC42 in cochlear hair cells leads to enhanced stereocilia developmental deficits. In conclusion, the present data suggest that CDC42 plays a pivotal role in regulating hair cell stereocilia development.

## Introduction

As the mechanosensitive receptor cells in the inner ear, hair cells are characterized by their hairy-looking hair bundles, which consist of hundreds of actin-based stereocilia and one microtubule-based kinocilium on the apical surface of each cell (Flock and Cheung, 1977). The kinocilium is important for hair bundle development as well as planar cell polarity (PCP) establishment, while the stereocilia are essential for mechano-electrical transduction (MET), the process that converts mechanical signals into electrical signals (Lindeman et al., 1971; Hudspeth and Jacobs, 1979; Jones et al., 2008). In each hair cell, the stereocilia are organized into several rows of increasing heights, forming a staircase-like pattern (Tilney et al., 1980). Deflection of stereocilia toward the taller row direction opens the MET channels at the tips of shorter row stereocilia, eventually leading to the influx of cations into hair cells (Hudspeth and Jacobs, 1979; Beurg et al., 2009).

The actin core of stereocilia consists of a bundle of cross-linked actin filaments (F-actin), with their barbed (plus) ends pointing toward the distal tips (Flock and Cheung, 1977; Tilney et al., 1980). During development, the stereocilia start as short apical microvilli and develop into the final mature morphology by increasing the length and numbers of F-actin core (Tilney et al., 1992; Krey et al., 2020). In adults, the stereocilia are quite stable and actin polymerization/depolymerization is only detected at the distal tips (Zhang et al., 2012; Drummond et al., 2015; Narayanan et al., 2015). The development and maintenance of stereocilia is tightly regulated, and deficits in this process usually lead to hearing loss or balancing deficits (Ciuman, 2011; Barr-Gillespie, 2015). With the rapid progress of genetic, transcriptomic, and proteomic techniques, many proteins have been identified to participate in the development and/or maintenance of stereocilia (Barr-Gillespie, 2015; McGrath et al., 2017; Ellwanger et al., 2018; Krey and Barr-Gillespie, 2019; Velez-Ortega and Frolenkov, 2019).

The Rho GTPase cell division cycle 42 (CDC42) is a key regulator of the actin cytoskeleton (Sit and Manser, 2011). It has been long known that CDC42 stimulates Arp2/3-dependent actin polymerization and plays an important role in the formation of filopodia, another finger-like actin-based cell protrusion that is similar to stereocilia and microvilli (Rohatgi et al., 1999; Chen et al., 2000; Yang et al., 2006). Recently, Ueyama et al. (2014) reported that CDC42 localizes in the stereocilia of cochlear hair cells, and that *Cdc42* gene disruption causes deficits in stereocilia maintenance. In the present work, we further explore the role of CDC42 in stereocilia development using *Cdc42* conditional knockout mice and CDC42 inhibitor. Our present data suggest that CDC42 regulates hair cell stereocilia development both in a cell autonomous and non-autonomous manner.

## Materials and Methods

### Plasmids and Antibodies

Mouse cDNAs encoding wild-type or mutant CDC42 were subcloned into pEGFP-C2. Rabbit anti-CAPZB2 antibody (Cat. No. AB6017) was purchased from Merck, and its specificity has been validated previously (Avenarius et al., 2017). Rabbit anti-MYO15A antibody was described and validated previously (Zou et al., 2014). Mouse anti-EPS8 antibody (Cat. No. 610143) was purchased from BD Biosciences, and its specificity has been validated previously (Zampini et al., 2011). Other antibodies and additional reagents were as follows: mouse anti-GFP antibody (Abmart, Cat. No. M20004); Alexa Fluor 488-conjugated donkey anti-rabbit IgG (Thermo Fisher Scientific, Cat. No. A21206); Alexa Fluor 488-conjugated donkey anti-mouse IgG (Thermo Fisher Scientific, Cat. No. A21202); Alex Fluor 546-conjugated donkey anti-mouse IgG (Thermo Fisher Scientific, Cat. No. A10036); TRITC-conjugated phalloidin (Sigma-Aldrich, Cat. No. P1951); iFluor 405-conjugated phalloidin (Abcam, Cat. No. ab176752).

### Mice

All animal experiments were approved by the Animal Ethics Committee of Shandong University School of Life Sciences (Permit Number: SYDWLL-2020-31) and performed accordingly. *Cdc42^*loxP/*+^* mice were developed as previously reported (van Hengel et al., 2008). *Atoh*1^*Cre*⁣/ +^ knock-in mice that express Cre recombinase under the control of *Atoh1* promoter were developed as previously reported (Yang et al., 2010).

### Scanning Electron Microscopy and Stereocilia Length/Width Quantification

SEM was performed as previously described (Wang et al., 2017). Dissected mouse temporal bone was fixed with 2.5% glutaraldehyde in 0.1 M phosphate buffer overnight at 4°C. The cochleae were then taken out and post-fixed with 1% osmium tetroxide in 0.1 M phosphate buffer at 4°C for 2 h. After dehydration in ethanol and critically point drying using a Leica EM CPD300 (Leica, Germany), samples were mounted and sputter coated with platinum (15 nm) using a Cressington 108 sputter coater (Cressington, United Kingdom). Images were taken using a Quanta250 field-emission scanning electron microscope (FEI, Netherlands) with a beam strength of 5 kV.

Stereocilia length quantification were performed as previously described (Li et al., 2020). Briefly, SEM images were taken in two different imaging planes with a known angle between them, and the relative stereocilia projection length and its angles in the reconstructed three-dimensional coordinates were measured using Photoshop. The stereocilia length was then calculated using the equations mentioned in the above report. Stereocilia width quantification were performed as previously described (McGrath et al., 2021). Briefly, stereocilia with approximately perpendicular orientation in SEM images were used for calculations. A line was drawn perpendicularly across the stereocilium at approximately the same distance above the taper region of each measured stereocilium. The length of this line was then measured using Image J to give rise to the width of the stereocilium.

### Whole-Mount Immunostaining

All steps were performed at room temperature unless otherwise indicated. The auditory sensory epithelia were dissected out of the temporal bone and fixed with 4% paraformaldehyde (PFA) in PBS for 20 min, followed by permeabilization and blocking with PBT1 (0.1% Triton X-100, 1% BSA, and 5% heat-inactivated goat serum in PBS, pH 7.3) for 40 min. After that, the samples were incubated with primary antibody in PBT1 at 4°C overnight, followed by sequential incubation with secondary antibody in PBT2 (0.1% Triton X-100 and 0.1% BSA in PBS) for 2 h and phalloidin in PBS for 30 min. The samples were mounted in PBS/glycerol (1:1), and images were taken using a confocal microscope with a 1.4 NA/100 × Kort M27 objective lens (LSM 700, Zeiss, Germany).

### Injectoporation

Injectoporation was performed as previously described (Du et al., 2020). Briefly, the cochlear sensory epithelia were isolated from P2 mice and cultured in DMEM/F12 with 1.5 μg/ml ampicillin. Expression plasmids (0.2 μg/μl in Hanks’ balanced salt solution) were delivered to hair cells using a glass pipette of 2 μm tip diameter. A series of three pulses at 60 V lasting 15 ms at 1-s intervals were applied by an electroporator (ECM Gemini X2, BTX, CA). The tissues were cultured for 24 h *in vitro* and then incubated with phalloidin in PBS for 30 min. The samples were mounted in PBS/glycerol (1:1), and images were taken using a confocal microscope with a 1.4 NA/63 × Kort M27 objective lens (LSM 900, Zeiss, Germany).

### Protein Purification and Localization in Permeabilized Hair Cells

Protein expression and purification was performed as previously described (Cao et al., 2013). Briefly, the coding sequence encoding wild-type or mutant CDC42 fused to EGFP was inserted into expression vector pET-28a, which was then transformed into *Escherichia coli* BL21(DE3) cells. His-tagged EGFP-CDC42 protein was induced in the presence of 0.4 mM isopropyl-β-d-thiogalactopyranoside (IPTG) at 16°C, then purified using Ni-NTA agarose (TransGen) according to manufacturer’s instructions.

The subcellular localization of purified protein in permeabilized hair cells was examined as described before (McGrath et al., 2021). All steps were performed at room temperature unless otherwise indicated. Briefly, the cochlear epithelia were dissected out of temporal bone in Hank’s balanced salt solution, followed by incubation with 40 μg purified EGFP-CDC42 protein in cytoskeletal buffer (20 mM HEPES, pH 7.5, 138 mM KCl, 4 mM MgCl_2_, 3 mM EGTA, 1% bovine serum albumin, 0.05% saponin, and 2 mM fresh ATP) for 5 min. Samples were then fixed with 4% PFA for 30 min, followed by blocking with 0.1 M PBS containing 5% (v/v) donkey serum for an hour. Afterward, samples were incubated with mouse anti-GFP antibody in 0.1 M PBS containing 5% (v/v) donkey serum for an hour, then Alex Fluor 546-conjugated donkey anti-mouse IgG in PBST buffer (0.01% Triton X-100 and 0.1% BSA in PBS) for an hour, followed by incubation with iFluor 405-conjugaetd phalloidin in PBS for 30 min. After mounting in PBS/glycerol (1:1), samples were imaged with a confocal microscope with a 1.4 NA/63× Kort M27 objective lens (LSM 900, Zeiss, Germany).

### Statistical Analysis

All experiments were performed at least three times independently. The numbers of analyzed animals or cells are indicated in the figures or figure legends. Data were shown as means ± standard error of mean (SEM). Student’s two-tailed unpaired *t*-test was used to determine statistical significance, and *P* < 0.05 was considered statistically significant.

## Results

### Hair Cell-Specific *Cdc42* Inactivation Leads to Stereocilia Development Deficits

**FIGURE 1:**
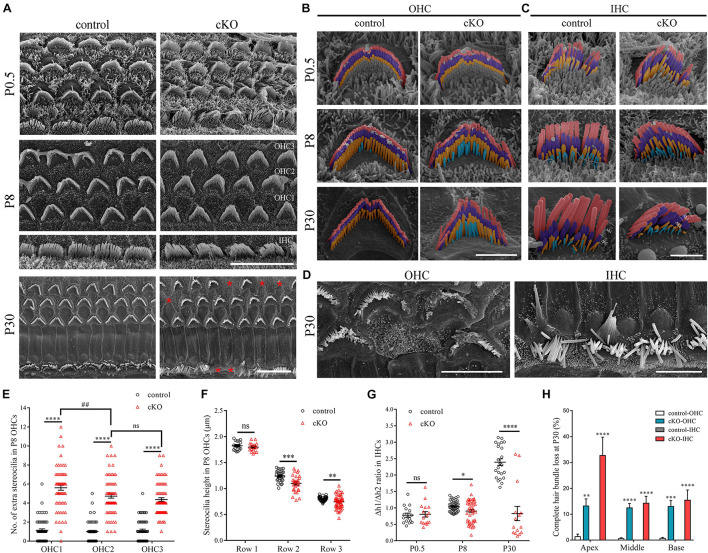
Hair cell-specific *Cdc42* inactivation leads to stereocilia development deficits. **(A)** Low magnification SEM images of the hair bundles from control or cKO mice at different ages as indicated. Shown are images taken from the middle turns. Asterisks indicate complete loss of hair bundles. Scale bar: 10 μm. **(B,C)** High magnification pseudo-colored SEM images of hair bundles from control or cKO OHCs **(B)** and IHCs **(C)** at different ages as indicated. The first, second, and third row stereocilia are indicated by pink, purple, and yellow, respectively. The extra fourth-row stereocilia are indicated by cyan. Δh1 and Δh2 represent the relative distance from 1st row stereocilium tip to its parallel connecting 2nd row stereocilium tip and the 2nd row stereocilium tip to its parallel connecting 3rd row stereocilium tip, respectively. Shown are images taken from the middle turns. Scale bars: 2 μm. **(D)** High magnification SEM images of severely disorganized hair bundles from control or cKO mice at P30. Shown are images taken from the middle turns. Scale bar: 5 μm. **(E)** Number of the extra fourth-row stereocilia per P8 OHC was analyzed according to the results from **(B)**. **(F)** Average stereocilia height in different rows of P8 OHCs were analyzed according to the results from **(B)**. **(G)** The Δh1/Δh2 ratio in IHCs at different ages was analyzed according to the results from **(C)**. **(H)** Percentage of P30 OHCs and IHCs with complete hair bundle loss was calculated according to the results from **(A)**. For statistical analyses in **(E–H)**, images were randomly taken from at least three animals for each group. The bars indicate mean ± SEM values. ^∗^*P* < 0.05; ^∗∗^*P* < 0.01; ^∗∗∗^*P* < 0.001; ^*⁣*⁣**^P < 0.0001; ^##^*P* < 0.01; ns, no significant difference.

To investigate the potential role of CDC42 in stereocilia development, we first crossed *Cdc42^*loxp/loxp*^* mice with *Atoh1^*Cre/*+^* knock-in mice that express Cre recombinase in developing cochlear hair cells from embryonic day 14.5 (E14.5) (Yang et al., 2010). The resultant *Atoh1^*Cre/*+^*;*Cdc42^*loxp/loxp*^* mice are referred to as *Cdc42* cKO mice thereafter in this work. *Atoh1^*Cre/*+^*;*Cdc42^*loxp/*+^* and *Cdc42^*loxp/loxp*^* mice were used as controls. Scanning electron microscopy (SEM) was then performed to examine the morphology of hair bundles in *Cdc42* cKO mice and control mice. At postnatal day 0.5 (P0.5), the morphology of hair bundle is largely indistinguishable between *Cdc42* cKO and control cochlear hair cells, with multiple rows of immature stereocilia protruding from the apical surface of hair cells (Figures 1A–C). By P8, most of the short-row immature stereocilia are resorbed and only 3 or 4 rows are remained in the control OHCs and IHCs, respectively (Figures 1B,C). However, in P8 *Cdc42* cKO OHCs, there are more fourth-row stereocilia remained, suggesting that immature stereocilia resorption is affected by *Cdc42* inactivation (Figures 1B,E). The extra fourth-row stereocilia in the *Cdc42* cKO OHCs persist when examined at P30, suggesting that it is a result of reduced stereocilia resorption instead of delayed stereocilia resorption (Figure 1B). Meanwhile, the stereocilia number in the regular rows is slightly decreased in the *Cdc42* cKO OHCs (Supplementary Figures 1A–C). By P30, stereocilia fusion, fragmentation, or even complete stereocilia loss could be observed in the *Cdc42* cKO OHCs and IHCs (Figures 1A,D,H).

The stereocilia height is also affected by *Cdc42* inactivation. By P8, there is significant variability of stereocilia height in the second and third rows in *Cdc42* cKO OHCs and IHCs, which becomes more apparent at P30 (Figures 1B,C,F). Moreover, in the *Cdc42* cKO OHCs, the average height of second- and third-row stereocilia is slightly decreased compared with control OHCs (Figures 1B,F). The height of IHC stereocilia is difficult to measure because of the imaging angle in most SEM images (Figure 1C). Nevertheless, the ratio of height difference between row 1 and row 2 stereocilia to that between row 2 and row 3 stereocilia is significantly decreased in the *Cdc42* cKO IHCs especially at P30 (Figures 1C,G).

**FIGURE 2:**
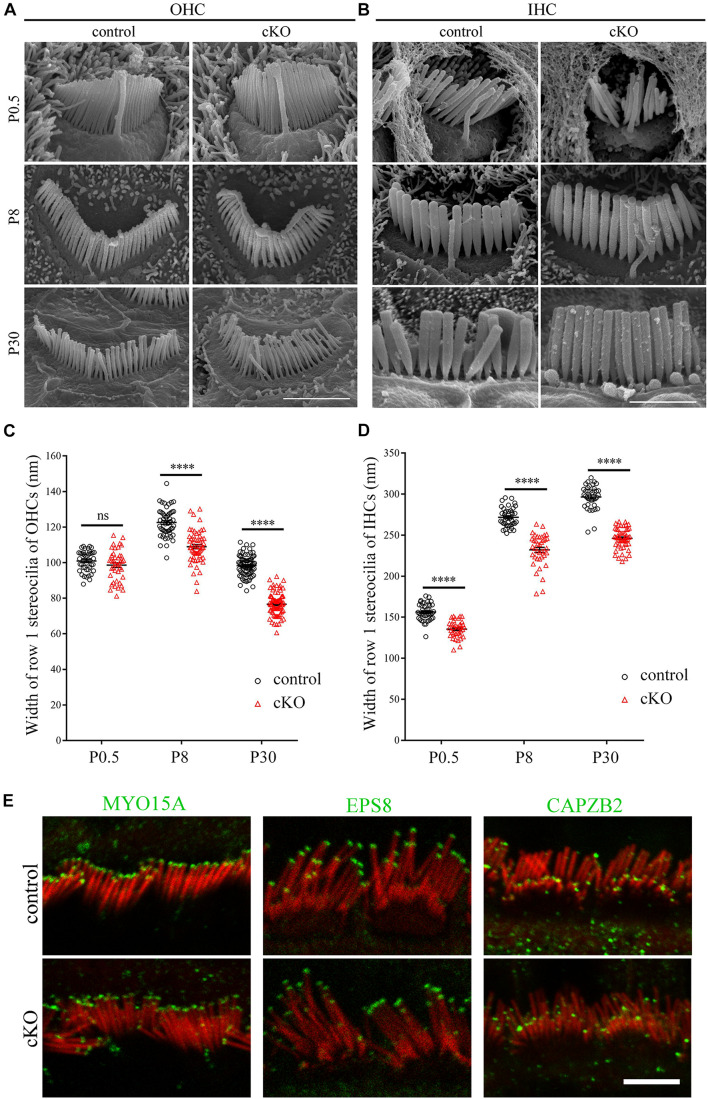
Hair cell-specific *Cdc42* inactivation leads to decreased stereocilia width. **(A,B)** High magnification SEM images of hair bundles (dorsal view) from OHCs **(A)** and IHCs **(B)** at different ages as indicated. Shown are images taken from the middle turns. Scale bars: 2 μm. **(C,D)** Width of the tallest-row stereocilia in OHCs **(C)** and IHCs **(D)** at different ages as indicated were analyzed according to the results from **(A,B)**, respectively. For statistical analyses in **(C,D)**, images were randomly taken from at least 3 animals for each group. The bars indicate mean ± SEM values. ****P < 0.0001; ns, no significant difference. **(E)** Whole-mount immunostaining showing localization of MYO15A, EPS8 and CAPZB2 in IHCs of P9 control or cKO mice. TRITC-phalloidin (red) was used to visualize the stereociliary F-actin core. All images were taken from the apical turns of cochlea using a confocal microscope. Scale bar: 5 μm.

Albeit the height of the tallest row stereocilia is largely unaffected in the *Cdc42* cKO hair cells, it seems that their width is decreased compared to control mice (Figures 1B,C). To examine the width of the tallest stereocilia more precisely, we took SEM images from the side of the tallest row stereocilia and quantified the stereocilia width. The results show that the width of the tallest-row stereocilia of *Cdc42* cKO OHCs is significantly decreased from P8 (Figures 2A,C). Similarly, the width of the tallest-row stereocilia of *Cdc42* cKO IHCs is significantly decreased from P0.5 (Figures 2B,D). Taken together, our present data suggest that CDC42 plays an important role in stereocilia development.

### Stereociliary Tip Localization of Row 1 and 2 Complex Components Is Not Affected in the *Cdc42* cKO Hair Cells

It has been known that stereocilia height is tightly regulated by the so-called row 1 and row 2 complex, which are localized at the tips of the highest-row stereocilia or shorter-row stereocilia, respectively (Krey et al., 2020; McGrath and Perrin, 2020). We then performed whole-mount immunostaining to examine whether *Cdc42* inactivation affects the stereociliary tip localization of known row 1 components MYO15A and EPS8 as well as row 2 component CAPZB2 (Belyantseva et al., 2003; Manor et al., 2011; Zampini et al., 2011; Avenarius et al., 2017). The results show that stereociliary tip localization of these proteins is not affected in the *Cdc42* cKO IHCs (Figure 2E) and OHCs (Supplementary Figure 2). Therefore, it seems that although *Cdc42* inactivation leads to stereocilia development deficits, it does not affect the general stereocilia row identity.

### CDC42 Inhibitor ML141 Treatment Leads to Stereocilia Development Deficits

**FIGURE 3:**
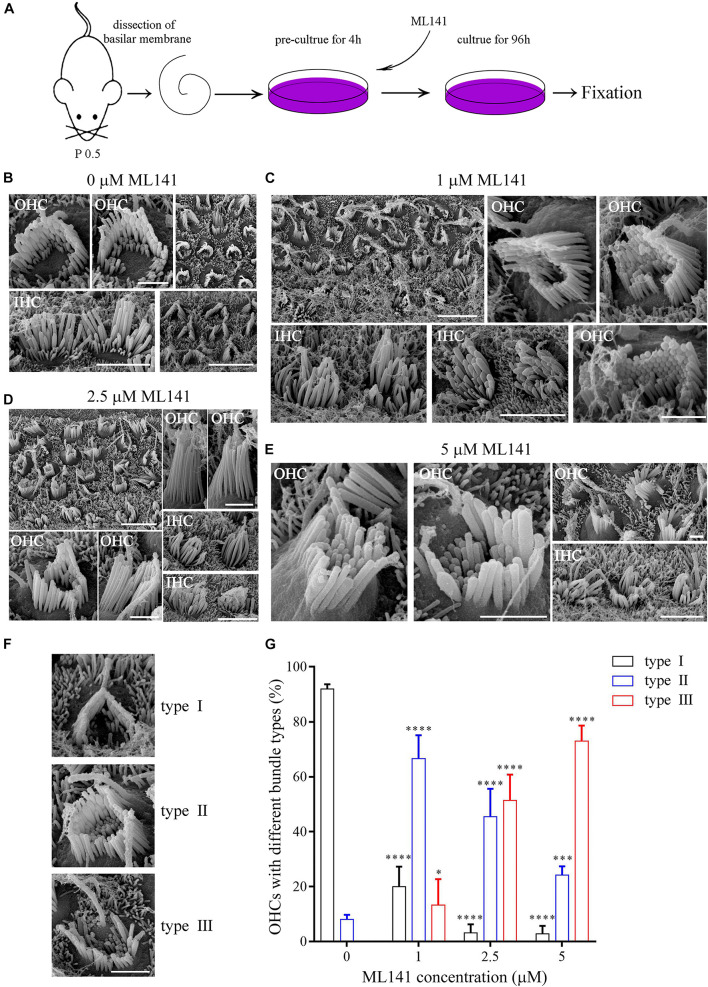
CDC42 inhibitor ML141 treatment leads to stereocilia development deficits. **(A)** Schematic drawing of the strategy of ML141 treatment and SEM imaging. **(B–E)** SEM images of hair bundles after treatment of ML141 at different concentrations as indicated. Scale bars: 5 μm in low magnification images; 1 μm in high magnification OHC images; 3 μm in high magnification IHC images. **(F)** Representative images showing three types of hair bundles with different PCP phenotypes. Scale bar: 1 μm. **(G)** Percentages of hair cells of different types were analyzed from three independent experiments. The bars indicate the mean ± SEM values. ^∗^*P* < 0.05; ^∗∗∗^*P* < 0.001; ^*⁣*⁣**^P < 0.0001.

We next examined the role of CDC42 in stereocilia development using a known CDC42 inhibitor ML141 (Surviladze et al., 2010). The cochlear sensory epithelia from P0.5 wild-type mice were cultured in presence of ML141 at 0, 1, 2.5, or 5 μM for 96 h, followed by fixation and SEM imaging (Figure 3A). The results show that 1 μM ML141 treatment leads to significant stereocilia disorganization, which is exaggerated at higher concentration of ML141 (Figures 3B–E). Noticeably, the staircase-like pattern of hair bundle is compromised, and more rows of stereocilia are observed in the ML141-treated hair cells (Figures 3B–E). Moreover, the PCP of hair cells is also significantly altered. We classified hair cells into three groups according to their PCP phenotypes, namely type I (roughly normal PCP), type II (circle-shaped stereocilia), and type III (reversed PCP) (Figure 3F). In the untreated controls, almost all hair cells are type I and only a small fraction of hair cells are type II (Figure 3G). However, in the presence of ML141, most hair cells show altered PCP in a dosage-dependent manner (Figure 3G). Taken together, our data show that stereocilia development is significantly affected by CDC42 inactivation either genetically or chemically.

### Constitutively Active Mutant CDC42 Leads to Enhanced Stereocilia Deficits

**FIGURE 4:**
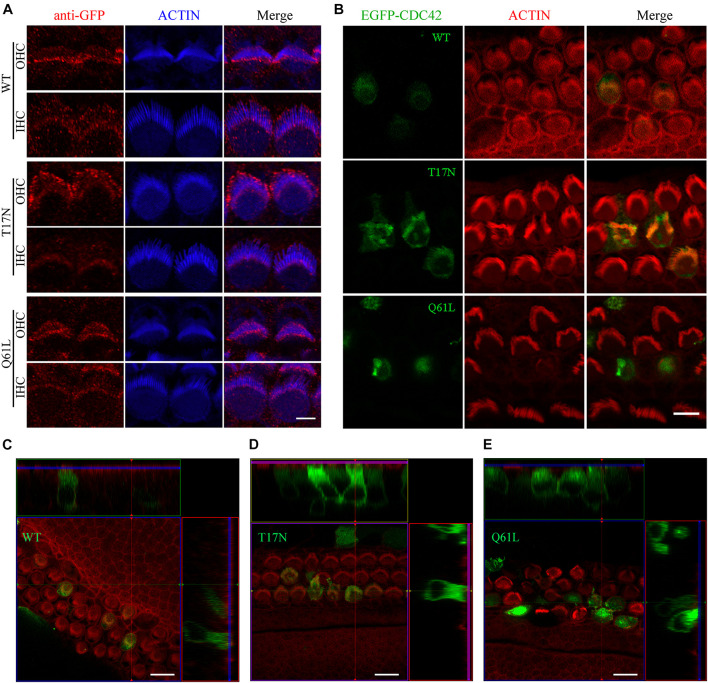
Constitutively active CDC42 mutant leads to enhanced stereocilia deficits. **(A)** Whole-mount staining showing localization of purified exogenous wild-type or mutant CDC42 proteins in saponin-permeabilized P0.5 OHCs and IHCs. iFluor 405-conjugated phalloidin was used to visualize stereociliary F-actin core. GFP antibody was used to amplify the signals of EGFP fused with CDC42 proteins. Scale bar: 2 μm. **(B)** Cochlear explants from P2 wild-type mice were injectoporated with expression vectors to express EGFP-tagged wild-type or mutant CDC42 in hair cells. TRITC-conjugated phalloidin was used to visualize stereociliary F-actin core. Scale bar: 5 μm. **(C–E)** 3D reconstruction showing the side view of hair cells injectoporated with different CDC42 constructs as indicated. Scale bar: 10 μm.

As a small Rho GTPase, CDC42 is reversibly cycling between an active (GTP-bound) and inactive (GDP-bound) state (Hart et al., 1991). We next wanted to examine the effect of CDC42 in different state on stereocilia. We made use of two point mutations that mimic CDC42 in different state, namely inactive CDC42 mutant (T17N) and constitutively active CDC42 mutant (Q61L) (Kozma et al., 1995). We first examined whether exogenous CDC42 mutants could target to the stereocilia following permeabilization of unfixed cochlear sensory epithelia with 0.05% saponin (McGrath et al., 2021). The results show that wild-type CDC42 and two CDC42 mutants all localize in the stereocilia of P0.5 mice, with enrichment at the stereociliary tips (Figure 4A). However, all three forms of CDC42 are mainly detected in the cell body of saponin-permeabilized P6 cochlear hair cells, suggesting that CDC42 may show dynamic subcellular localization in the hair cells during development (Supplementary Figures 3A–C).

We then examined the effect of different CDC42 mutants on stereocilia development by employing the injectoporation assay and confocal microscopy. CDC42-expression vectors were injectoporated into P2 wild-type cochlear sensory epithelia, followed by *in vitro* culture for 24 h and confocal imaging. Stereociliary F-actin core was visualized using phalloidin. The results show that wild-type and CDC42 T17N mutant localize in the stereocilia as well as cell body of injectoporated hair cells, with largely normal stereocilia morphology (Figures 4B–D). In contrast, CDC42 Q61L mutant injectoporation results in complete stereocilia loss in most hair cells (21 out of 24 analyzed cells), suggesting that this constitutively active mutant CDC42 leads to enhanced stereocilia deficits (Figures 4B,E).

## Discussion

It was recently reported that the Rho GTPase CDC42 is localized in the stereocilia of cochlear hair cells, and that cochlear hair cell stereocilia of *Atoh1-Cre*; *Cdc42^*flox/flox*^* mice develop normally but progressively degenerate after maturation, suggesting that CDC42 plays an important role in stereocilia maintenance but not development (Ueyama et al., 2014). In the present work, however, we show that CDC42 is indispensable for normal stereocilia development in cochlear hair cells. One possible reason for this discrepancy might be the different *Atoh1-Cre* mouse lines used in these two works. Ueyama and colleagues used transgenic *Atoh1-Cre* mice (Matei et al., 2005), whereas we used *Atoh1-Cre* knock-in mice (Yang et al., 2010). Therefore, different Cre recombinase activity in these two mouse lines might account for the slightly different stereocilia phenotypes. Another possibility is that the subtle stereocilia development deficits revealed in the present work might have simply been overlooked in the earlier study.

Our SEM results reveal that the morphology of cochlear hair cell stereocilia is largely indistinguishable between P0.5 *Cdc42* cKO mice and control mice. Stereocilia deficits are apparent in P8 *Cdc42* cKO cochlear hair cells, which include increased stereocilia height variation and decreased stereocilia width. Moreover, the height difference between rows is significantly altered in IHCs, and extra shortest row stereocilia exist in OHCs of *Cdc42* cKO mice. Despite of the stereocilia deficits, the stereocilia row identity largely remains normal, given that the stereociliary tip localization of row 1 complex components MYO15A and EPS8 as well as row 2 complex component CAPZB2 is unchanged at this age. Moreover, CDC42 inhibitor ML141 causes similar stereocilia development deficits plus PCP phenotypes, further supporting an important role of CDC42 in stereocilia development.

Stereocilia development is a multi-step, tightly controlled process (Tilney et al., 1992; Kaltenbach et al., 1994). In immature cochlear hair cells, there are multiple rows of elongated stereocilia on the apical surface, among which the shorter rows are eventually resorbed, leaving 3–4 rows of stereocilia grow to their final height in the mature mammalian cochlear hair cells (Kaltenbach et al., 1994). The underlying mechanism of shorter row stereocilia resorption largely remains unknown. Our present data suggest that CDC42 might play a role in this process. The extra fourth-row stereocilia in *Cdc42* cKO OHCs is possibly a result of reduced stereocilia resorption, which might be explained by altered actin polymerization and stabilization of stereocilia due to *Cdc42* inactivation. Similar reason might also account for the observed deficits in stereocilia length and width of *Cdc42* cKO cochlear hair cells.

Noticeably, our present data suggest that appropriate CDC42 activity is important for stereocilia development. It has been reported that in cultured cells, overexpression of constitutively active CDC42 mutant (Q61L) promotes the formation of filopodia (Kozma et al., 1995). In line with this, gain of CDC42 activity by disrupting ARHGAP1 (previously known as CDC42GAP) also promotes filopodia formation (Yang et al., 2006). However, our present data show that overexpression of CDC42 Q61L mutant in cochlear hair cells leads to complete loss of stereocilia. This is in sharp contrast to the reports in filopodia formation. It is possible that stereocilia development and/or maintenance is regulated so tightly that imbalance of this process would inevitably cause stereocilia deficits. Similar scenario is also observed in DIA1, whose dysfunction is associated with autosomal dominant sensorineural hearing loss DFNA1 (Lynch et al., 1997). Constitutive activation of DIA1 causes stereocilia disorganization as well as hearing loss in mice (Ueyama et al., 2016; Ninoyu et al., 2020).

Our present data show that treatment with CDC42 inhibitor results in more severe stereocilia phenotypes than hair cell-specific *Cdc42* inactivation. Noticeably, apparent PCP deficits are observed in cochlear culture treated with CDC42 inhibitor, but not in the hair cell-specific *Cdc42* cKO mice, suggesting that other cells such as supporting cells are involved in CDC42-mediated PCP regulation. This is consistent with previous reports that dysregulated PCP is observed in mice deleting *Cdc42* in both hair cells and supporting cells, but not in mice deleting *Cdc42* only in hair cells (Ueyama et al., 2014; Kirjavainen et al., 2015). In line with this, *in situ* hybridization reveals that *Cdc42* transcripts are expressed ubiquitously in the cochlea (Anttonen et al., 2012). The ubiquitous expression of *Cdc42* in the inner ear is also supported by RNAseq results of embryonic and adult mice (Cai et al., 2015; Scheffer et al., 2015; Liu et al., 2018). Taken together, the present data suggest that CDC42 regulates stereocilia development both in a cell autonomous and non-autonomous manner.

Loss of another Rho GTPase RAC1 has also been shown to cause stereocilia development deficits, such as stereocilia flattening, fragmentation, as well as altered PCP (Grimsley-Myers et al., 2009). Different stereocilia phenotypes observed in *Cdc42* and *Rac1* knockout mice suggest that these two Rho GTPases play important and non-complementary roles in stereocilia development. Guanine nucleotide exchange factor (GEF) ARHGEF6 specifically activates CDC42 and RAC1, and loss of ARHGEF6 causes stereocilia deficits (Zhu et al., 2018). Moreover, p21-activated kinase 1 (PAK1), a downstream effector of CDC42 and RAC1, is required for stereocilia development (Cheng et al., 2021). These studies highlight the essential role of Rho GTPases in stereocilia development. At present, the precise localization of these proteins in the stereocilia remains unknown, which prevents us from fully understanding the underlying mechanism. New imaging methods such as super-resolution microscopy definitely will help to address this question (Liu et al., 2019; Qi et al., 2019, 2020).

## Data Availability Statement

The original contributions presented in the study are included in the article/Supplementary Material, further inquiries can be directed to the corresponding author/s.

## Ethics Statement

The animal study was reviewed and approved by the Animal Ethics Committee of Shandong University School of Life Sciences (Permit Number: SYDWLL-2020-31).

## Author Contributions

ZX: study concept and design. HD, HZ, YS, and XZ: acquisition of data. HD, ZC, YW, and ZX: analysis and interpretation of data. YW and ZX: supervision. HD, YW, and ZX: drafting the manuscript. All authors contributed to the article and approved the submitted version.

## Conflict of Interest

The authors declare that the research was conducted in the absence of any commercial or financial relationships that could be construed as a potential conflict of interest.

## Publisher’s Note

All claims expressed in this article are solely those of the authors and do not necessarily represent those of their affiliated organizations, or those of the publisher, the editors and the reviewers. Any product that may be evaluated in this article, or claim that may be made by its manufacturer, is not guaranteed or endorsed by the publisher.
