# Supplementary material for: The Rho GTPase Cell Division Cycle 42 Regulates Stereocilia Development in Cochlear Hair Cells
Source: Front Cell Dev Biol. 2021 Oct 22;9:765559. doi: 10.3389/fcell.2021.765559 (PMC8570139; doi:10.3389/fcell.2021.765559)
Supplement: Supplementary file 1 [file Data_Sheet_1.PDF]

The Rho GTPase CDC42 regulates stereocilia development in cochlear hair cells

Haibo Du[1], Hao Zhou[1], Yixiao Sun[1], Xiaoyan Zhai[1], Zhengjun Chen[2,3], Yanfei Wang[1*], Zhigang Xu[1,4*]

[1]Shandong Provincial Key Laboratory of Animal Cell and Developmental Biology, School of Life Sciences, Shandong University, Qingdao, Shandong 266237, China

[2]State Key Laboratory of Cell Biology, Shanghai Institute of Biochemistry and Cell Biology, Center for Excellence in Molecular Cell Science, Chinese Academy of Sciences (CAS), Shanghai 200031, China

[3]School of Life Science and Technology, ShanghaiTech University, Shanghai 200031, China

[4]Shandong Provincial Collaborative Innovation Center of Cell Biology, Shandong Normal University, Jinan, Shandong 250014, China

[*]Correspondence

Yanfei Wang, Shandong Provincial Key Laboratory of Animal Cell and Developmental Biology, School of Life Sciences, Shandong University, Qingdao, Shandong 266237, China. E-mail: wang_yf@sdu.edu.cn (Y. W.)

Zhigang Xu, Shandong Provincial Key Laboratory of Animal Cell and Developmental Biology, School of Life Sciences, Shandong University, Qingdao, Shandong 266237, China. E-mail: xuzg@sdu.edu.cn (Z. X.)

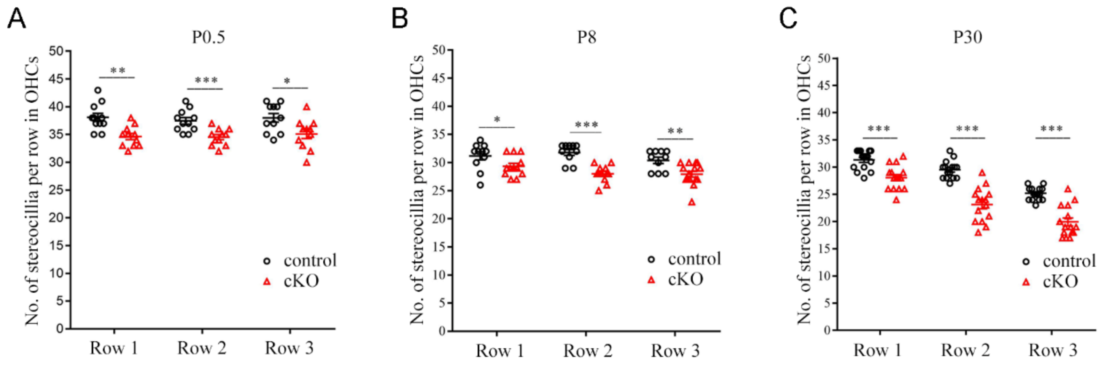

Supplementary Figure 1. Stereocilia number per row is decreased in *Cdc42* cKO OHCs. Stereocilia number per row in OHCs of control or *cdc42* cKO mice at P0.5 (A), P8 (B) and P30 (C) was analyzed according to the SEM results, which were randomly taken from at least 3 animals for each group. The bars indicate mean ± SEM values. *, P < 0.05; **, P < 0.01; ***, P < 0.001.

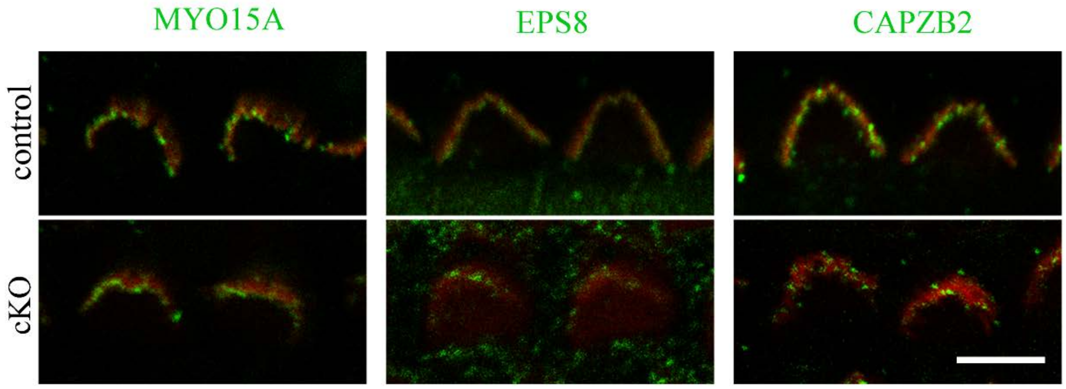

Supplementary Figure 2. Stereociliary tip localization of MYO15A, EPS8, and CAPZB2 is unaffected by *Cdc42* inactivation. Whole-mount immunostaining showing localization of MYO15A, EPS8 and CAPZB2 in OHCs of P9 control or cKO mice. TRITC-phalloidin (red) was used to visualize the stereociliary F-actin core. All images were taken from the apical turns of cochlea using a confocal microscope. Scale bar: 5 μm.

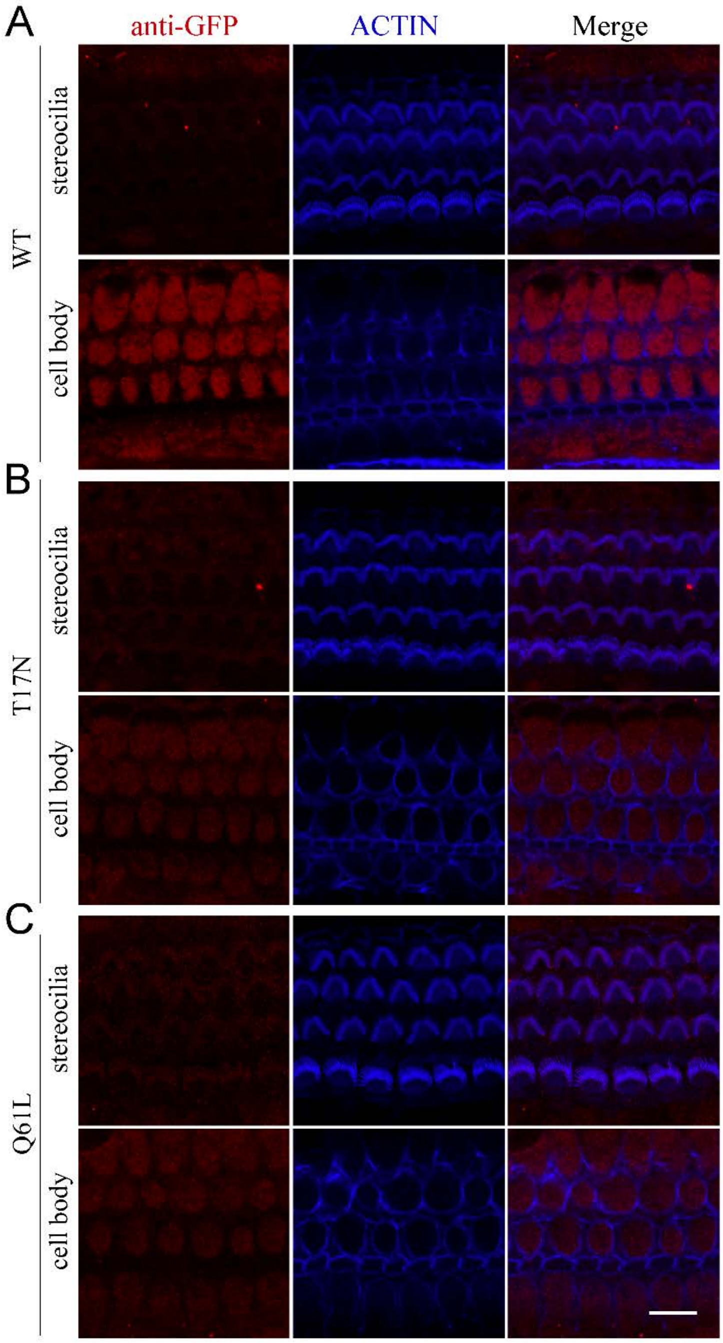

Supplementary Figure 3. Exogenous CDC42 is mainly localized in the cell body of saponin-permeabilized P6 cochlear hair cells. Whole-mount staining showing localization of purified exogenous wild-type (A), T17N (B), and Q61L (C) CDC42 proteins in saponin-permeabilized P6 cochlear hair cells. iFluor 405-conjugated phalloidin was used to visualize stereociliary F-actin core. GFP antibody was employed to amplify the signals of EGFP-fused CDC42 proteins. Scale bar: 10 μm.
